# New Avenues for Parkinson’s Disease Therapeutics: Disease-Modifying Strategies Based on the Gut Microbiota

**DOI:** 10.3390/biom11030433

**Published:** 2021-03-15

**Authors:** Marina Lorente-Picón, Ariadna Laguna

**Affiliations:** Neurodegenerative Diseases Research Group, Vall d’Hebron Research Institute (VHIR)-Center for Networked Biomedical Research on Neurodegenerative Diseases (CIBERNED), Passeig Vall d’Hebron 119-129, 08035 Barcelona, Spain; marina.lorente@vhir.org

**Keywords:** Parkinson’s disease, gut microbiota, therapeutic modulation, gut–brain axis, prebiotics, probiotics, antibiotics, synbiotics, Mediterranean diet, fecal transplants, live biotherapeutic products

## Abstract

Parkinson’s disease (PD) is a multifactorial neurodegenerative disorder that currently affects 1% of the population over the age of 60 years, and for which no disease-modifying treatments exist. Neurodegeneration and neuropathology in different brain areas are manifested as both motor and non-motor symptoms in patients. Recent interest in the gut–brain axis has led to increasing research into the gut microbiota changes in PD patients and their impact on disease pathophysiology. As evidence is piling up on the effects of gut microbiota in disease development and progression, another front of action has opened up in relation to the potential usage of microbiota-based therapeutic strategies in treating gastrointestinal alterations and possibly also motor symptoms in PD. This review provides status on the different strategies that are in the front line (i.e., antibiotics; probiotics; prebiotics; synbiotics; dietary interventions; fecal microbiota transplantation, live biotherapeutic products), and discusses the opportunities and challenges the field of microbiome research in PD is facing.

## 1. Introduction

Parkinson’s disease (PD) is the second most common chronic, progressive neurodegenerative disorder after Alzheimer’s disease (AD), affecting 1% of the population over the age of 60 years [1,2]. PD is currently affecting nearly seven to ten million people worldwide and the incidence and prevalence of PD is expected to increase substantially in the next decades due to the increase in life expectancy that our society has experienced [3].

The hallmark clinical symptom of PD is motor impairment (i.e., rigidity, movement slowness, resting tremor, among others [4,5,6]), which is first noticeable when there is already 40–60% neuronal loss in the substantia nigra (SN) [7,8]. However, clinical features include non-motor symptoms such as depression [9], REM-sleep behavior disorder [10,11], autonomic dysfunction [12], olfactory disruption [13], and constipation [14,15], among others. In the past 15 years, non-motor PD symptoms have raised a lot of attention because they can occur years before the onset of motor symptoms [16,17]. Thus, they are considered as valuable markers of early disease stages [18]. More specifically, gastrointestinal (GI) dysfunctions have been well described and their frequency is very high in PD. It mostly appears at premotor stages of the disease, although is also prevalent later on and tends to get worse as the disease progresses [19,20,21]. Some of the common symptoms are sialorrhea, dysphagia, gastroparesis, bowel dysfunctions and constipation, the last one being the most prevalent [14,15,22,23,24].

From a neuropathological point of view, PD is characterized by the progressive and irreversible loss of dopaminergic neurons present in the SN pars compacta (SNpc) and their axon projections causing the loss of the nigrostriatal neuronal pathway and triggering dopamine depletion in the basal ganglia [25,26,27]. Neuronal degeneration in other brain regions has also been reported [28,29,30], preferentially occurring within neurons that contain the dark-brown cytoplasmic pigment neuromelanin (NM) [29,31]. PD neuropathology is also characterized by the presence of cytoplasmic inclusions enriched in aggregated forms of α-synuclein (α-syn) called Lewy bodies (LB) [32]. However, the molecular and cellular processes that trigger the misfolding, fibrillization, LB formation, and spread of α-syn in the brain remain poorly understood [33].

Although there is extensive research on the pathophysiological mechanisms involved in PD, it is still not clear what is or are the initial triggers. Disease-relevant pathway identification is essential to define candidates for the development of disease-modifying therapies [33,34,35]. The current treatment remains symptomatic and yet far from modifying disease onset or early progression. The most commonly used treatments are dopamine agonists like Levodopa (L-dopa) for dopamine replacement therapy and resulting in an improvement of motor symptoms in the early stage of the disease [36]. However, they do not influence the rate of clinical disease progression and eventually they cause serious side effects such as motor complications and dyskinesias. L-dopa is usually administered in combination with an inhibitor of its peripheral conversion to dopamine (carbidopa or benserazide) [37]. In addition, PD is characterized by impairment of other pathways targeting disturbances of the serotonergic, noradrenergic, glutamatergic, γ -aminobutyric acid (GABA)ergic, and cholinergic systems, which are not targeted by L-dopa [38]. There are other treatment options that don’t use dopamine agonists such as deep brain stimulation, which consists of the placement of high frequency stimulating electrodes in the region of the ventral intermediate nucleus of the thalamus and stimulation of the subthalamic nucleus or the internal globus pallidus to reduce tremor and also decrease bradykinesia, rigidity, and gait impairment [39,40]. Further research on pathophysiology-based therapeutic interventions able to slow or halt disease progression is needed.

The GI tract in humans is one of the largest interfaces between the host and the environment. Inside the GI tract, a complex ecological community called the gut microbiota exists and is composed by more than 100 trillion of microorganisms of several species [41]. It offers many benefits to the host through a wide range of physiological functions such as nutrients and metabolites absorption and synthesis [42], protection against pathogens [43], and regulation of host immunity [44], as well as integrity of the intestinal epithelium. The gut microbiota is established at birth and keeps developing and fluctuating in composition until the adult life, where it is found to be relatively stable [45]. However, stress, antibiotics, environmental factors, infections, diet, lifestyle, and geography are factors known to influence, alter and modify gut microbiota composition [46].

Although there is still no consensus on the composition of a healthy gut microbiota, it seems that the more diverse and balanced the gut microbiota is, the more it has the ability to resist perturbations or to recover towards a healthy state more easily [47]. Studies of non-targeted sequencing combining metagenomics and 16S rRNA gene amplicon provided the first insights into the taxonomic composition of gut microbiota [43]. The dominant gut bacterial phyla are *Actinobacteria, Proteobacteria, Fusobacteria, Verrucomicrobia, Firmicutes* and *Bacteroidetes*, with the last two phyla representing 90% of gut microbiota [48,49,50]. On the other hand, it has been shown that alterations in gut microbiota composition and function can lead to dysbiosis, which has been linked to the etiology of multiples GI [51] and non-GI diseases such as anxiety, depression [52], autism [53], spectrum disorders [54], AD [55], and for the interest of this review, PD [24].

Given its extensive and multifunctional role in the GI tract and other parts of the host, the gut microbiota has emerged as a new attractive target for potential therapeutic approaches. The focus of this review is to present in a comprehensive way the currently available evidence of therapeutic strategies modulating the gut microbiota in PD, and discuss the limitations and expectations this emerging field of research is facing.

## 2. Evidences for an Altered Gut–Brain Axis in Parkinson’s Disease

The gut–brain axis refers to the bi-directional communication between the central nervous system (CNS) and the enteric nervous system (ENS) [56]. In a broader sense, it includes the neuroendocrine and neuroimmune systems, including the hypothalamic–pituitary–adrenal axis (HPA axis), sympathetic and parasympathetic arms of the autonomic nervous system (ANS), including the ENS and the vagus nerve, and the gut microbiota [57,58]. There is increasing evidence suggesting that gut microbiota is involved in signaling mechanisms that affect CNS neuronal circuits, thus being a modulator of brain physiology, function, communication, and even behavior. The gut microbiota includes bacteria, archaea, fungi, and viruses. However, evidence from comparative analyses of archea, fungi, and viruses in PD are limited. A recent report on the gut mycobiome in PD reported no differences in the fungi abundance between patients and controls [59], and another reported decreased total virus abundance in PD patients [60]. Thus, further studies are necessary to evaluate the contribution of archea/fungi/virus to PD. On the contrary, several recent and extensive reviews describe the different evidence suggesting that gut bacteria are involved in the pathophysiology of PD [61,62,63]. We do not aim to go through the same aspects but just to contextualize and briefly summarize the main findings that support the bacterial community in gut microbiota as a target for therapeutic development in PD.

### 2.1. Neuropathological, Epidemiological, and Experimental Animal Model Evidence

PD is a multifactorial and multisystemic disease. Epidemiological and experimental evidence suggest that environmental factors might have an important role in triggering or propagating the pathology [64,65]. Several pieces of evidence have led to the hypothesis that GI dysfunction is a contributor to PD, the GI tract being in close connection to the environment and a gateway system to the brain [21,66].

Firstly, Braak et al. proposed that the pathological process of protein aggregation begins in the olfactory nucleus and in the enteric nerve cell plexuses. Moreover, they suggested an active retrograde transport of α-syn from the ENS to the CNS via the dorsal motor nucleus of the vagus nerve (DMNV) [67,68]. This hypothesis is supported by data from rodent and non-human primate experimental models [69], where different forms of α-syn were transported via the vagus nerve and reached the DMNV in the brainstem after injection into the intestinal wall [66,70,71,72,73,74]. Moreover, it has been proposed that α-syn is capable of self-propagating in a prion-like manner transferring the pathology to unaffected cells by promoting misfolding of the normal α-syn [75,76,77,78,79]. Emerging evidence has shown that protein nucleation and aggregation may be influenced by an extracellular amyloid protein called “curli” secreted by *Escherichia Coli* [80] inducing neuronal deposition of a-syn in the gut and promoting neuroinflammation. However, even if there is evidence arguing in favor of a gut–brain direction in terms of α-syn pathology spreading, there are different studies highlighting the possibility that GI α-syn aggregates might have a brain origin [81,82,83]. Both possibilities are not mutually exclusive and they are still extensively debated [62,84,85,86,87,88], while more research is needed for a definitive proof and to better understand the biochemical and molecular mechanisms involved.

Secondly, there is epidemiological evidence from a Danish study reporting that full truncal vagotomy decreased the risk of developing PD [89], although they did not differentiate truncal from selective vagotomy. More recently, another study in the Swedish population suggested that truncal vagotomy could lower the risk of PD but not selective vagotomy [90]. These data also suggested that the vagus nerve may be involved in the pathogenesis of PD.

Thirdly, data from experimental animal models support the idea that gut dysbiosis, caused by intestinal infection or environmental toxins, among other possible causes, may act as a triggering event in PD development and/or progression An in vivo study using α-syn overexpressing mice reported that gut microbiota is involved in motor impairments and GI dysfunction via postnatal gut–brain signaling by microbial molecules that impact microglia activation, neuroinflammation, and α-syn aggregation [91]. Interestingly, they also showed that gut microbiota transplants from PD patients into α-syn overexpressing mice promoted motor impairments compared with gut microbiota transplants from healthy controls (HC), suggesting that specific gut microbiota species may be implicated in PD rather than general microbial stimulation, and that alterations in gut microbiota represent a risk factor for PD [91]. Consistent with these findings, another study also showed that fecal microbiota transplants (FMT) from 1-methyl-4-phenyl-1,2,3,6-tetrahydropyridine (MPTP) treated PD mice into wild type mice induced impaired motor function and decreased striatal dopamine [92]. Finally, another study using mice lacking PINK1 demonstrated that intestinal infection with Gram-negative bacteria caused a decrease in the density of dopaminergic axonal varicosities in the striatum and impairment in some motor functions [93].

### 2.2. Metagenomic Evidence

The use of shotgun metagenomics to carry out non-targeted sequencing of the gut microbiota community and the use of 16S rRNA gene amplicon surveys to study the bacterial and archaeal community structures have become popular and accessible techniques for clinical researchers [94]. These have facilitated studies in human cohorts to investigate the associations between gut microbiota dysbiosis, disruption of gut homeostasis, and CNS-related diseases such as PD [24,60,95,96,97,98,99,100,101,102,103,104,105,106,107,108,109,110,111,112]. Despite our knowledge has increased substantially in the past five years, the functional implications of the altered gut microbiota in the onset or progression of PD remain to be fully elucidated. Boertien et al. extensively reviewed the available fecal gut microbiome composition studies in PD human patients and compared them based on their methodologies and results [49]. They identified 16 case-control studies concerning original gut microbiome data, and they agreed in the pathological imbalance between the microbial community in PD patients compared with HC. Additional studies [111,113,114,115,116,117,118,119] have been published since then and we have summarized all findings in Table 1. At the phylum level, the increase of *Proteobacteria, Actinobacteria*, and *Verrucomicrobia* was reported in 4, 5, and 6 studies, respectively (Table 1). At the family level, a decrease in the relative abundances of *Prevotellaceace* and *Lachnospiraceae* was reported in 5 and 9 studies, respectively (Table 1), and an increase in *Lactobacillaceae, Bifidobacteriaceae*, *Enterobacteriaceae* and *Verrucomicrobiaceae* was consistently reported in 5, 5, 6, and 8 studies, respectively (Table 1). At the genus level, a decrease in the relative abundance of *Blautia, Roseburia and Faecalibacterium* was reported in 6, 10, and 10 studies, respectively (Table 1), in addition to the increase of *Lactobacillus* and *Akkermansia*, was reported in 5 and 13 studies, respectively (Table 1). All of these studies confirm that the gut microbiota is altered in PD.

Each group of bacteria in the gut has its own role and they produce small molecules and metabolites that can have a positive or a negative impact on the host health [120]. Interestingly, some of these metabolites and molecules seem to be modulators of PD pathogenesis. *Prevotellaceace* and *Lachnospiraceae* family members, as well as the genus *Blautia, Roseburia*, and *Faecalibacterium*, which are reduced in PD, are commensals involved in the mucus formation of the gut mucosal layer and in the production of short chain fatty acids (SCFAs) [109,121,122]. SCFAs, the major ones being butyrate, propionate, and acetate, are microbial metabolites that constitute the major products from bacterial fermentation of dietary fibers in the intestines [123,124] and are considered key candidate mediators [125]. They have the ability to directly affect GI physiology and the intestinal barrier function, as well as to keep digestive structures in optimal condition and have a role in peristalsis [126,127]. Moreover, they induce the expression of anti-inflammatory cytokines and suppress the expression of the pro-inflamatory ones [128,129]. The reduction observed in PD may affect gut permeability and cause local and systemic susceptibility to the presence of bacterial antigens and endotoxins due to the disruption of the gut mucus, which can be an environmental trigger of PD. Correlating with gut microbiota reductions, fecal SCFA concentrations are also reduced in PD patients compared to HC [97]. 

The gut *Verrucomicrobiaceae* family and *Akkermansia* genus are beneficial bacteria that reduce gut barrier disruption and control gut permeability to maintain the integrity of the intestinal barrier, although their role in neuron degeneration remains unclear [109]. In particular, *Akkermansia muciniphila* (*A. muciniphila*), a gram-negative bacteria located mainly in the mucus layer of the intestinal epithelium and producing mucin-degrading enzymes, that has an important role in maintaining intestinal barrier homeostasis and exerts competitive inhibition on other pathogenic bacteria that degrade the mucin [130,131]. Its abundance in the human intestinal tract is inversely correlated to several disease states such as obesity [132,133,134], while an increase of the relative abundance is consistently reported in PD patients [49,135]. *A. muciniphila* is capable of inducing a wide range of immune-modulatory responses in vitro including induction of cytokine production and activation of Toll-like receptors 2 and 4 (TLR2 and TLR4) [136], indicating that it cannot be strictly defined as anti- or pro- inflammatory, but may instead have a more complex role in preserving the balance of the immune gut microenvironment. Nevertheless, an increased abundance of genus *Akkermansia* and an increased intestinal permeability in PD may expose the intestinal neural plexus directly to oxidative stress or toxins [114]. Thus, even if there is evidence that its presence is beneficial for normal gut function, and it is being considered a good probiotic [137,138], the maintenance of steady state levels of Akkermansia may be a pre-requisite for gut homeostasis.

*Enterobacteriaceae* are a large family of gram-negative bacteria residing in the gut at low levels and localized closely to the mucosal epithelium [139]. This family is among the most commonly overgrown bacteria in many conditions involving gut inflammation [140,141] as they are responsible for the production of the endotoxins lipopolysaccharides (LPS) [142]. The presence of LPS disrupts gut homeostasis inducing the production of pro-inflammatory cytokines that may produce an inflammatory response in the CNS [143]. 

The increased levels of the families *Lactobacillaceae* and *Bifidobacteriaceae* in PD seem ironical, as they are usually well recognized as probiotics [23,144], and they are used in some clinical trials to treat constipation [145] and to reduce bloating and abdominal pain [146]. In addition, they increased the expression of tight junction proteins and upregulated mucus secretion [147]. However, even if they seem beneficial for the healthy population, they may act as opportunistic pathogens and cause infection in immune-compromised individuals [111,148]. 

Despite all the accumulated evidence, several inconsistencies exist between metagenomic studies that could be due to different experimental designs (i.e., fecal sampling, DNA extraction protocols, targeted 16S marker gene regions, statistical analysis) and/or patient enrollment criteria (i.e., ethnic origins, host genetics, geography, diet, lifestyle, and other confounding factors). These discrepancies make it difficult to reach a general agreement and determine which is the PD dysbiotic profile and which are the exact alterations in metabolic pathways [49,149].

## 3. Important Co-Factors for Gut–Brain Disturbances in PD

Although not the scope of this review, understanding gut pathophysiology in the context of PD becomes fundamental for the development of pathophysiology-based therapeutic approaches. With this section, we aim at highlighting some of the key factors. 

### 3.1. Aging

One of the main risk factors for PD is aging [150]. Age has been demonstrated to have an impact in human gut microbiota where an increase in microbiota’s diversity is seen from infants to adults followed by a decrease in biodiversity as adults age [151,152,153,154]. Elder people have different gut microbiota compared with healthy adults and this difference can be attributed to several causes such as lifestyle, medications, infections, organ dysfunctions, diseases, and other related factors [45,155]. The changes at the phylum-level composition are characterized by the decrease in *Firmicutes* and *Actinobacteria*, and there is an increase in *Bacteroidetes* and *Proteobacteria* [156]. Moreover, changes in gut microbiota diversity due to aging are linked to loss of intestinal barrier integrity that can lead to leakage of bacterial antigens and release of LPS, SCFA, or neurotransmitters. This contributes to the deterioration of the neuro-immune system in a process called immunosenescence [157] and is directly related with inflammaging [158,159]. How aging and inflammaging contribute to PD development and vice-versa is still largely unknown. 

### 3.2. Neuroinflammation

PD is not considered an immune disease, but inflammation and neuroinflammation are important players in the etiology and/or the progression of the disease [160,161]. Post-mortem analyses of human PD patients and experimental animal studies indicate that activation of glial cells and the increase of pro-inflammatory factor levels are common features of the PD brain [162,163,164]. Chronic release of pro-inflammatory cytokines such as tumor necrosis factor-α (TNF-α), interleukin-1β (IL-1β), and interferon-gamma (IFN-γ) by activated astrocytes and microglia leads to the aggravation of dopaminergic neurons’ degeneration in the SNpc [165,166,167]. In addition, infiltration and accumulation of peripheral immune cells are also found in affected brain regions in PD patients [168]. Interestingly, some studies report alterations in the intestinal epithelial barrier [169,170] and the presence of chronic intestinal inflammation in some PD patients [171,172] leading to the hypothesis that intestinal and peripheral inflammation contribute to neuroinflammation aggravating the progression of the disease [173].

Gut microbiota activity is completely linked to the status of the intestinal immune system [174]. Under healthy conditions, gut microbiota contributes to the maintenance of the intestinal epithelial barrier integrity through tight junctions between cells. In this way, microbes are allowed to persist in the intestine and execute their symbiotic functions without causing inflammation and at the same time providing the host with a physical barrier to protect from pathogens and to preserve homeostasis [175]. However, the introduction of external substances or pathogens and gut dysbiosis can both lead to increased gut inflammation, thus affecting intestinal homeostasis and altering CNS function via the release of neurotransmitters and cytokines [173,176,177]. Toll-Like receptors (TLR) are innate immune receptors expressed in intestinal epithelial cells, ENS cells and microglia [178] that play a fundamental role in promoting the release of pro-inflammatory cytokines in response to various components from bacteria including peptidoglycans and lipoproteins (TLR2) and LPS on the cell surface of Gram-negative bacteria (TLR4) [179]. Evidence from human studies reporting that TLR2 and TLR4 expression is increased in blood and brain samples of PD patients [180], and from animal studies in TLR4-KO mice treated with rotenone [172], strengthen the important role that TLR4 may play in intestinal and brain inflammation. Another set of key mediators in the brain–gut immune communication are SCFA, which have anti-oxidant and anti-inflammatory properties that may affect the intestinal mucosal immunity, the peripheral immune system and also modulate central neuroimmune brain function [121,181]. Moreover, they serve as energy substrate for intestinal epithelial cells [182] and have also been shown to reinforce the integrity of the blood–brain barrier (BBB) in mice [183]. One study reported low levels of SCFAs and high levels of calprotectin in stool samples from PD patients [184], which was associated with disruption of the intestinal barrier integrity. However, a recent study found no significant correlations between abundance of SCFAs and immune or permeability-related factors in stool or plasma, raising doubts about the association between SCFAs production and inflammation [184]. Additionally, another study showed in mice that, despite the overall protective function of SCFAs, they might have the potential to regulate autoimmune CNS inflammation both positively and negatively [185]. For further information on this controversy, please refer to reviews discussing specifically the role of SCFAs in inflammation regulation [125,186].

### 3.3. Genetics

Genetic mutations are also linked to PD development although only 5–10% of patients are inherited with a monogenic form of PD [187]. Genome-wide association studies (GWAS) have identified autosomal recessive mutations in *DJ-1*, *PINK1*, *PARK7*, and *Parkin*, as well as autosomal dominant mutations in *SNCA*, *LRRK2*, and *GBA* [188]. Despite *LRRK2* mutations being the most common cause of dominant PD, less than 30% of the carriers will end up developing the disease [189,190,191]. The variable penetrance suggests that other factors besides the mutation itself, i.e., genetic, epigenetic, or environmental, might have a role in modifying vulnerability of the different central and autonomic systems. The gut microbiota could be one of the factors that modulate the manifestation of symptoms in genetic PD forms.

Interestingly, an in vitro study reported that G2019S mutation in *LRRK2* causes gene expression changes in intestinal epithelial cells that may result in GI impairment [192]. In addition, an in vivo study revealed that LRRK2 is expressed by enteric neurons of the small intestine, also pointing to the possible involvement of LRRK2 in the regulation of ENS [193]. LRRK2 is highly expressed in both innate and adaptive immune cells, supporting the idea of its central role in immune responses [194]. Thus, LRRK2 might regulate inflammatory responses in the intestine and mutations may have multiple downstream effects [195]. For example, it has been implicated in the secretion of important anti-microbial components in response to enteric bacterial pathogens [196]. On the other hand, LRRK2 has been reported to modulate susceptibility to various infections caused for instance by *Mycobacterium tuberculosis, Salmonella typhimurium, and Listeria monocytogenes* [197,198,199,200]. Hence, *LRRK2* mutations can modify inflammatory responses after exposure to certain pathogens, potentially modulating disease development and/or progression in some individuals, which could partially explain the incomplete penetrance for *LRRK2* mutations [35,201].

## 4. Targeting the Gut–Brain Axis in PD

Because of the accumulating evidence from human and animal studies showing gut microbiota alterations in PD, several research groups are working in the identification of specific microbes and the pathways that connect them to the brain. Considering how much easier it is to manipulate the gut than the brain, the possibility to modulate the manifestation of PD symptoms by changing the gut microbiota is attracting a lot of attention both from the academic and pharmaceutical sectors. Much work is still needed given the complexity of the gut–brain axis, but many believe in the therapeutic potential of gut microbiota, and evidence is slowly coming out.

### 4.1. Antibiotics

Antibiotics are chemical substances that at low concentrations can inhibit or eliminate some microorganisms. They have attracted a lot of interest because some have other biological actions in the CNS that are independent from their anti-microbial activity such as anti-inflammatory, neuroprotective, or α-syn anti-aggregation effects [202,203,204]. Those characteristics can be of crucial importance to design PD or other neurodegenerative diseases’ treatments. The new challenge is to uncover the mechanisms of these compounds and elucidate how they interact with CNS function. Since many are already approved by regulatory agencies for use in humans, the process of getting into clinical trials would be significantly accelerated [205,206]. However, the usage of antibiotics can alter the relative abundance of bacterial species causing the disappearance of some and the appearance and growth of new bacterial species, thus essentially leading to dysbiosis [207]. Thus, it is important to understand the biology, the properties, and the relations between the coexisting species and how they interact with the host to be able to design new therapeutic approaches while preserving and protecting the beneficial bacteria of our gut microbiota [208].

Minocycline has been proposed as a potential therapeutic agent against PD as it exerts anti-inflammatory, anti-apoptotic, and antioxidant effects [209]. In one study, minocycline showed neuroprotective activity in dopaminergic neurons against MPTP neurotoxicity in vivo, as well as it preserved dopamine levels in the striatum and nucleus Accumbens. This effect was associated with reductions in inducible nitric oxide synthase (iNOs) and caspase-1 [210]. Another study also reported that minocycline prevented MPTP-induced activation of microglia, the formation of IL-1β, and the activation of NADPH–oxidase and iNOS [211]. The neuroprotective effects of minocycline were also shown in vitro against the NO-induced phosphorylation of p38 Mitogen-Activated Protein Kinase (MAPK) [210] as well as the 6-hydroxydopamine (6-OHDA)-induced cell toxicity [212]. Together, the available studies suggest that minocycline inhibits microglial activation through p38 MAPK pathway, which regulates the release of pro-inflammatory factors from activated microglia [213,214]. Conversely, Diguet et al. demonstrated enhanced toxicity of MPTP in monkeys treated with minocycline [215]. Despite the controversy, minocycline was moved as a potential drug candidate to treat PD into a Phase II clinical trial in 2006 and 2008, which unfortunately did not show benefit. Moreover, they suggest that minocycline may be of concern when considering a long-term study [216,217].

Doxycycline (DOX) is also an inexpensive antibiotic with a broad therapeutic spectrum and exceptional bioavailability [202]. Recent in vitro studies have shown that DOX induced the reshaping of α-syn oligomers so that they did not evolve into fibrils, thus affecting α-syn ability to destabilize biological membranes, cell viability, and seeding capacity [218]. In an in vivo mouse PD model, DOX blocked 6-OHDA neurotoxicity by reducing microglial and astrocyte activation through inhibition of iNOs [219]. Moreover, another study also reported neuroprotective effects of DOX on dopaminergic neurons in an MPTP toxicity model by suppressing microglial activation and astrogliosis. In this case, the authors propose that the effect is due to anti-apoptotic and anti-inflammatory mechanisms involving downregulation of matrix metalloproteinase (MMP)-3 [220]. Similarly, another study in an LPS rat model of PD demonstrated that DOX had a neuroprotective effect on dopaminergic neurons by downregulating the expression of microglial major histocompatibility complex II (MHC II) [221].

Ceftriaxone (CTX), a widely prescribed β-lactam antibiotic, is another possible target for several neurodegenerative disorders. CTX is known to increase glutamate transporter subtype 1 (GLT-1) expression and could thus be a feasible venue to increase glutamate uptake and reduce excitotoxicity [222]. In vivo studies proved that CTX increased GLT-1 expression and glutamate uptake, attenuated tyrosine hydroxilase loss in a calcium-dependent manner, and attenuated locomotor behavior deficits associated with a 6-OHDA-induced lesion [223]. More recently, the same group reported that a CTX regime after a 6-OHDA-induced lesion but prior to and during L-DOPA treatment reduced dyskinesia severity [224]. Moreover, CTX treatment prevented motor, working memory, and object recognition impairments due to MPTP-induced toxicity in a rat model of PD [225]. CTX has also been reported to have antioxidant and anti-inflammatory effects. A study showed how CTX attenuated pro-inflammatory cytokines such as tumor necrosis factor alpha (TNF-α) and IL-β in striatum region; in addition, it diminished the oxidative injury and restored the levels of endogenous antioxidant enzymes in an MPTP PD rat model [226]. Interestingly, an in vitro study showed that CTX binds to α-syn with good affinity and blocks its polymerization [227].

Rifampicin, a semi-synthetic macrocyclic antibiotic [228], has also shown to counterbalance key pathological characteristics of PD (Table 2). In vitro, it binds α-syn and inhibits its fibrillation by stabilizing the monomeric form of the protein as well as disaggregates the existing pre-formed fibrils [229]. Moreover, pre-treatment of PC12 cells prior to 1-Methyl-4-phenyl pyridinium (MPP+) intoxication increased cell viability and prevented α-syn aggregation [230]. Similar neuroprotective effects of rifampicin against MPP+ intoxication have been shown in dopaminergic neurons [231]. A more recent study reported that rifampicin upregulated the expression of the glucose-regulated protein 78 (GRP78), a chaperone that is a hallmark of the unfolded protein response (UPR), via the PERK-eIF2α-ATF4 pathway to protect PC12 cells against rotenone-induced cytotoxicity [232]. Other studies reported anti-inflammatory properties of rifampicin. One study reported that rifampicin inhibited the production of proinflammatory factors such as iNOs, IL-1β, and TNF-α by downregulating nuclear factor-kappa B (NF-kB) and MAPK pathway from microglial cells [233]. More recently, another study showed that rifampicin pre-treatment exerts neuroprotection against rotenone-induced microglia inflammation in BV2 and SH-SY5Y cells [234]. In vivo studies showed that rifampicin attenuated neurodegeneration of the nigrostriatal dopaminergic pathway induced by MPTP toxicity in mice through regulation of oxidative stress [235].

### 4.2. Probiotics

Probiotics are live microorganisms that, when administered in adequate amounts, confer a health benefit to the host [236]. Increasing evidence supports the idea of using certain probiotics to modulate gut microbiota and its functions in order to prevent dysbiosis or to have a positive impact into the host health. Most of the commercially available probiotics contain *Lactobacillus*, *Bifidobacterium*, or *Saccharomyces* spp. [23].

Several studies in vitro and in vivo with animal models and humans’ preclinical trials demonstrated the potential benefits of probiotics in the prevention or treatment of GI disorders such as chronic inflammation or inflammatory bowel disease [237,238,239]. Interestingly, in the last 10 years, several studies reported that probiotics also have an influence in the CNS by showing efficacy in improving psychiatric disorder behaviors such as depression, anxiety or cognitive symptoms [240,241,242]. This evidence encouraged the field to test them for PD as they might be a powerful tool to modulate PD associated dysbiosis and improve GI dysfunction [63,243]. However, pre-clinical or clinical evidence on the beneficial effects of probiotics in PD is still very limited.

A recent in vitro study focused on assessing the effects of probiotics on samples from PD patients compared to HC [244]. They evaluated reactive oxygen species (ROS) and cytokines released by peripheral blood mononuclear cells (PBMCs) isolated from patients. They reported that, in PBMCs from PD and HC patients, *Lactobacillus salivarius* and *Lactobacillus acidophilus* showed the best results in decreasing the pro-inflammatory cytokines and increasing the anti-inflammatory ones, as well as in reducing ROS production. In addition, they showed that *Bifidobacterium breve*, *Lactobacillus plantarum*, and *Lactobacillus rhamnosus* significantly restored the integrity of the previously stressed (with a combination of TNF-α and IL1-ß) epithelial Caco-2 cells. Interestingly, these last two strains also exerted a robust capacity of inhibiting the growth of potential pathogen bacteria such as *Escherichia coli* and *Klebsiella*. However, in vivo longitudinal studies should be carried out to further support their beneficial effects [244]. Another in vivo study in a *Caenorhabditis elegans* (*C. elegans*) model of synucleinopathy revealed that treatment with a *Bacillus subtilis* probiotic inhibited α-syn aggregation and cleared preformed aggregates. They showed that the probiotic triggered the protective effect via spores and vegetative cells, partly due to a biofilm formation in the gut of worms and to the release of bacterial metabolites. Moreover, a *Bacillus subtilis* supplement altered the lipid composition of the cell, directly affecting α-syn aggregation [245]. Another study in a mouse PD model tested the neuroprotective effects of probiotics against MPTP and rotenone-induced toxicity. They fed animals for a month with an oral probiotic mixture containing *Lacobacillus rhamnosus GG, Bifidobacterium animalis lactis*, and *Lactobacillus acidophilus,* and, after 30 days, mice received MPTP injections. Results showed that *Lactobacillus rhamnosus* GG had a major role in preventing neurodegeneration of dopaminergic cells by upregulating neurotrophic factors such as brain-derived neurotrophic factor (BDNF) and Glial cell-derived neurotrophic factor (GDNF). Moreover, they reported downregulation of monoamine Oxidase-B (MAO-B) [246] expression, thus potentiating striatal neuronal responses to dopamine [247]. The exact mechanisms of the beneficial effects observed are not yet fully understood, but it could be associated with the fact that *Lacobacillus rhamnosus GG* may potently stimulate the growth of butyrate-producing strains [246]. In addition, another study used the same neurotoxic model feeding the animals with *Lacobacillus plantarum* PS128 by oral gavage during 28 days and injecting MPTP the last four days. Results showed amelioration in MPTP-induced motor deficits and dopaminergic neuronal death, together with attenuated MPTP-induced oxidative stress and neuroinflammation in the nigrostriatal pathway [248]. Furthermore, another recent study used the probiotic *Clostridium butyricum* in the MPTP model, where mice received MPTP injections for seven days and then were treated with the probiotic for four weeks. Results demonstrated improved motor deficits, attenuated dopaminergic neuron loss, improved synaptic dysfunction, and microglia activation in treated mice. They associated the neuroprotective mechanism with the glucagon-like peptide-1 (GLP-1/GLP-1 receptor) pathway [249].

In 2011, Cassani et al. proved that regular intake of fermented milk containing *Lactobacillus casei Shirota* improved stool consistency and reduced bloating and abdominal pain in PD patients suffering from constipation [145]. In addition, another randomized, double-blind, placebo-controlled clinical trial in 2016 also showed that regular intake of fermented milk containing multiple strains of probiotics (*Streptococcus salivarius, subsp. Thermophilus, Enterococcus faecium, Lactobacillus rhamnosus GG, Lactobacillus acidophilus, Lactobacillus plantarum, Lactobacillus paracasei, Lactobacillus delbrueckii, subsp. Bulgaricus and Bifidobacterium*) and prebiotic fibers improved constipation symptoms in PD patients in comparison with the placebo group [250]. In the same line, another study provided tablets, instead of using dairy products, containing two lactic bacteria, *Lactobacillus acidophilus and Bifidobacterium infantis*, for three months to randomly selected PD patients suffering from GI non-motor symptoms. They showed that probiotics were capable of improving abdominal pain and bloating in PD patients with lower GI non-motor symptoms [146]. More recently, Tan et al. proved that PD patients taking one capsule containing multi-strain probiotics (*Lactobacillus acidophilus, Lactobacillus reuteri, Lactobacillus gasseri, Lactobacillus rhamnosus, Bifidobacterium bifidum, Bifidobacterium longum, Enterococcus faecalis, Enterococcus faecium*) once a day for four weeks improved the number of spontaneous bowel movements in comparison with the placebo group [251].

Evidence seems to demonstrate that probiotic intake can improve non-motor symptoms related with GI dysfunction. However, there is less evidence of their efficacy in preventing or modulating motor PD symptoms. An in vivo study using transgenic MitoPark PD mice showed that administration of probiotics for 16 weeks (*Bifidobacterium bifidum, Bifidobacterium longum, Lactobacillus rhamnosis, Lactobacillus rhamnosus GG, Lactobacillus plantarum and Lactococcus lactis*) attenuated the motor impairment of this PD model. Moreover, the administration of probiotics had neuroprotective effects on dopaminergic cells preventing them from cell death [252]. These data correlate with a randomized, double-blind, placebo-controlled clinical trial that was conducted to evaluate movement and metabolic responses of PD patients to three months of probiotic intake. Patients were provided with capsules containing *Lactobacillus acidophilus, Bifidobacterium bifigum, Lactobacillus reuteri* and *Lactobacillus fermentum.* Results showed favorable impact on MDS-UPDRS (Movement Disorders Society-Unified Parkinson’s Disease Rating Scale) score but just few changes on the metabolic profiles (i.e., high sensitivity C-reactive protein (hs-CRP), blood glutathione, malondialdehyde, and insulin metabolism) [253].

To summarize, although additional studies are required before definite conclusions can be made, probiotics have multiple health benefits to the host and have an encouraging potential as preventive and therapeutic leads to restoring gut microbiota and the gut physiological function, and eventually also brain function, in PD patients. Current limitations to bear in mind are: (i) their effect varies depending on genera and species of the microorganism [254]; (ii) the mechanisms underlying their effects and the synergies that might occur between them still need to be elucidated. Hence, long term carefully designed clinical trials are required to obtain definite proof of the clinical efficacy of those probiotics in PD patients [23]. Metagenomic, metatranscriptomic, and metabolomics will be very valuable tools to globally examine interactions between probiotics, intestinal microbes, and the mammalian GI tract [255].

#### Probiotics and L-dopa

L-dopa is the current most widely used anti-Parkinsonian treatment that helps to mitigate motor PD symptoms [256]. Despite its extensive use, it has been associated with many side effects such as dyskinesia [257] and its efficacy varies highly across patients and across time. L-dopa crosses the BBB and is decarboxylated by the aromatic amino acid decarboxylase (AADC) enzyme to produce dopamine [256]. L-dopa is administered orally, so its bioavailability depends on the degree of metabolic processing by intestinal and hepatic enzymes before it reaches the brain [258]. Thus, it is usually given in combination with an AADC inhibitor such as carbidopa in order to minimize its peripheral metabolism [259] and ensure that sufficient L-dopa will reach the brain. However, pharmacokinetics of L-dopa has been found to be highly variable between patients and its efficacy decreases over time of treatment, and sometimes it is even ineffective in some patients [260].

It is known that the gut microbiota can influence the metabolism of different drugs, in many cases changing their efficacy and/or side effect profiles [258]. Thus, it has been hypothesized that L-dopa can also be metabolized by the gut microbiota using an alternative metabolic pathway and potentially reducing its bioavailability and leading to side effects [261,262]. A study reported that the tyrosine decarboxylase (tyrDC) enzyme produced by small intestinal bacteria from the genera *Enterococcus* and *Lactobacillus* [263,264] is responsible for the decarboxylation of L-dopa into dopamine in the gut. Interestingly, the authors showed that *Enterococcus faecalis* compromised L-dopa uptake and did not present susceptibility to carbidopa treatment. In addition, they reported that the relative abundance of *tyrDC* gene in stool samples from PD patients was positively correlated with higher daily L-dopa dosage requirement [265]. Related with this study, a different group described another possible pathway for gut bacterial L-dopa metabolism. They showed that tyrDC from *Enterococcus faecalis* converted L-dopa to dopamine and that a single-nucleotide polymorphism (SNP) of dopamine dehydroxylases (dadh) from *Eggerthella lenta*, subsequently metabolized dopamine into m-tyramine, thus reducing dopamine bioavailability. Interestingly, they found that abundance of *Enterococcus faecalis* and tyrDC correlated with metabolic status in fecal PD patients’ samples who had varying levels of L-dopa [266]. This data raised the possibility that levels of *Enterococcus faecalis* and tyrDC could be used as peripheral biomarkers in order to improve the regime of L-dopa treatment in PD patients. In addition, they found a stool compound, (S)-α-fluoromethyltyrosine (AFMT), that could prevent L-dopa decarboxylation and increase bioavailability by inhibiting tyrDC [266]. Importantly, some *Enterococcus* strains are included nowadays as food supplements in several probiotics preparations [75,267], and this should be considered in the management of PD patients.

More recently, one study investigated the effects of intraduodenal infusion of levodopa-carbidopa intestinal gel (LCIG) compared to L-dopa administration in gut microbiota composition. Interestingly, LCIG was related with an increased relative abundance of the *Enterobacteriaceae* family and the genus *Escherichia* and *Serratia* compared with the L-dopa group [268], suggesting that the pharmacological treatment used influences the gut microbiota composition of PD patients.

Finally, lower absorption of L-dopa has been reported in PD patients infected by *Helicobacter pylori* [269], suggesting that eradication of *Helicobacter pylori* by the aid of some probiotics might be useful in these patients (Table 3). Taken together, these studies highlight the importance of knowing the gut microbiota status of PD patients and of choosing between the different available pharmacological treatment options in order to achieve the best disease management possible.

### 4.3. Prebiotics

Prebiotics are non-digestible food ingredients that beneficially affect the host’s health by selectively stimulating the growth and/or activity of some genera of microorganisms [270,271]. Prebiotic definitions are generally attributed to dietary fibers (oligo and polysaccharides substrates) that are really important as most gut microbiota degrade dietary fibers to obtain energy for their own growth [272]. For instance, they are the main source of energy for *Bifidobacterium* and *Lactobacillus* [273]. However, as the complexity and function of gut microbial ecosystems is being unveiled, new microbial groups or species of interest for health purposes are being identified, and new research is also focused on the use of fructans, galacto-oligosaccharides, and lactulose for their benefits into the gut microbiota [270]. Prebiotics largely impact the composition of the gut microbiota and its metabolic activity, thus improving stool quality, reducing gut infections, improving bowel motility and general well-being [274,275]. Hence, it is relevant to study the beneficial effects they might have in GI dysfunction related with inflammatory processes and constipation [243] and that directly affect PD patients gut microbiota.

In PD, lower abundance of SCFA butyrate-producing bacteria could be corrected by the use of prebiotic fibers and in turn impact the regulation of inflammatory processes, gut barrier integrity, and peristalsis. In addition, butyrate can alter gene expression by inhibition of the chromatin-remodeling activity of histone deacetylases (HDAC) [276]. An in vitro study showed that treating dopaminergic neurons with sodium butyrate could rescue WT α-syn expressing cells from DNA damage, possibly by restoring the expression of DNA-repair genes [277]. An in vivo study demonstrated that HDAC inhibitors like sodium butyrate could ameliorate locomotor impairment in a Drosophila rotenone-induced PD model [278]. In addition, another study in a transgenic mouse model of diffuse LBs disease showed that long-term administration of phenylbutyrate increased DJ-1 activity. By upregulating DJ-1 activity, they showed reduced α-syn aggregation in the brain and prevented age-related motor impairment and cognitive function [279]. The above-mentioned studies used oral administration of sodium butyrate; because sodium butyrate is mostly absorbed in upper segments of the GI tract, the administration route led to an increase in plasma concentration that might well result in direct actions in the brain. On the other hand, butyrate produced by gut microbiota from the fermentation of prebiotic dietary fibers is considered to act locally in the large intestine, where it could promote both localized and systemic effects as another promising approach in the management of PD [274,280]. However, it is yet unclear which way for increasing butyrate levels would be best, hence preclinical studies are needed to evaluate how gut-derived butyrate affects PD pathophysiology. Apart from SCFA, another recent study used polymannuronic acid administration daily for four weeks in a MPTP-mouse model of PD and reported improved motor functions and dopaminergic neuroprotection. Treated mice also presented enhanced levels of homovanillic acid (HVA), serotonin (5-HT), 5-hydroxyindole acetic acid (5-HIAA) and GABA in comparison with MPTP mice [281], suggesting that prebiotics might also have beneficial effects on other neurotransmitter systems apart from the dopaminergic system.

### 4.4. Synbiotics

Synbiotics are described to be a combination of synergistically acting probiotics and prebiotics, where a prebiotic component selectively favors the metabolism or growth of a probiotic microorganisms, thus providing a beneficial effect to the host’s health [282,283]. Synbiotics should be created in appropriate combination in order to overcome possible difficulties in the survival of probiotics in the GI tract. Moreover, the combination of both should have a superior effect in the host health compared to the activity that they may have alone [275].

Evidence from a clinical study showed that consumption of symbiotic yogurt containing *Bifidobacterium animalis* and fructo-oligosaccharide as prebiotics increased bowel movements, stool quantity, and quality in a group of women with functional constipation in comparison with HC [284]. Another clinical study showed that supplementation of the combination of *Lactobacillus salivarius* and fructo-oligosaccharide reduced inflammation in healthy subjects, presenting better results than the subjects who just received supplementation with the probiotic [285]. In addition, a previously mentioned randomized, double-blind, placebo-controlled clinical trial also showed that regular intake of fermented milk containing multiple strains of probiotics and prebiotic fiber improved constipation symptoms in PD patients in comparison with placebo [250]. Another randomized and double-blind study showed that treatment with antibiotics followed by symbiotic supplementation of *Lactobacillus coagulan* and fructo-oligosaccharides in patients with small intestine bacteria overgrowth (SIBO), presented a better response to decreased abdominal pain, flatulence, and diarrhea than just the patients who received antibiotic treatment [286].

In conclusion, synbiotics are important as prebiotics support and complement probiotic action. However, it is still a big challenge to identify each prebiotic and its metabolites and how we can increase probiotics effectiveness, as co-metabolism exists. Moreover, data about the effect of synbiotics on metabolic health is limited, but it is probably associated with the independent studies done with probiotic and prebiotics, respectively. However, considering there is a huge number of possible combinations, the application of synbiotics for the modulation of gut microbiota in PD patients seems promising [243,275]; thus, further research should be supported.

### 4.5. Dietary Interventions

Our knowledge on the substances that can influence gut microbiota and their colonizing abilities has improved significantly in the past years. Therefore, another approach that can be used to modulate these microbial population is dietary intervention. Moreover, we can use specific nutrient combinations containing membrane phosphatide precursors such as uridine and docosahexaenoic acid (DHA) [287,288] together with cofactors, prebiotics, probiotics, or antibiotics in order to reduce barrier-related pathologies in the ENS and CNS, neuroinflammation, neurodegenerative processes, etc. [289]. This approach can also complement the traditional PD therapies and confer clinical benefits to PD patients since they might modulate motor and non-motor symptoms.

#### 4.5.1. Mediterranean Diet

Mediterranean diet, which is based in the daily consumption of vegetables, legumes, fruits, nuts, whole grains, and healthy fats, can beneficially impact the brain by multiple mechanisms. On the contrary, the Western diet is known for high amounts of fat and sugar and low intake of dietary fibers. Thus, the microbiome of people having a Mediterranean diet is characterized by the abundance of bacteria that uses dietary fiber to produce SCFA [290]. As mentioned previously, SCFA are the end-products of fermentation of non-digestible carbohydrates by gut microbiota, and they are really important for intestinal barrier function, gene expression, and mitochondrial function. In Western diets, where dietary fiber ingestion is low, the microbiota uses protein as an energy source [291]. SCFA-producing bacteria may be reduced and the growth of gram-negative bacteria may be favored, thus causing dysbiosis and an increase of LPS [292,293].

A study in idiopathic PD patients showed that some components of the Mediterranean diet were associated with slower PD progression while others were associated with rapid progression, thus providing evidence that targeted nutrition is associated with the rate of PD progression [294]. In another study, the association between the probability of prodromal PD in a Greek population-based cohort study of older adults and its possible association with Mediterranean diet adherence was investigated [295]. Probability of prodromal PD was calculated according to International Parkinson and Movement Disorder Society research criteria and a detailed food frequency questionnaire was used to evaluate dietary intake and calculate Mediterranean diet adherence score. Their results showed that adherence to the Mediterranean diet was associated with a lower probability of prodromal PD in older people. Moreover, a Swedish population-based cohort study investigated the association of adherence to a Mediterranean diet at middle age with the PD risk later in life, and showed an inverse association between both factors [296]. More recently, a German population-based cohort study tested whether a dietary intervention (i.e., ovo-lacto vegetarian diet including SCFA for 14 days) alone or combined with an additional physical colon cleaning (i.e., daily fecal enema over 8 days) may lead to changes of the gut microbiota in PD patients. Their results showed significant improvement in the Unified PD rating scale III (UPDRS III) score after the dietary intervention combined with fecal enema after a one-year follow-up [297]. Interestingly, another study showed neuroprotective effects of *Vitis vinifera* red grape seed and skin extract (GSSE) on midbrain dopaminergic neurons, both in vitro and in vivo, against 6-OHDA-induced toxicity [298]. In line with this, polyphenols, like GSSE, have gained great interest as they act as antioxidants and anti-inflammatory compounds [299]. However, research that addresses their influence on the gut microbiota is less developed than for other health benefits (i.e., cardiovascular risk) and the bioavailability of polyphenols is controversial since most polyphenols react with digestive enzymes under duodenal conditions and are metabolized into other molecules with unknown therapeutic potential [206].

To summarize, the Mediterranean diet is associated with a lower incidence and progression of PD, although further studies are needed to elucidate the potential causality of this association as well as the underlying neurobiological mechanisms. Still, one could speculate that the Mediterranean diet has a suggestive protective effect on PD risk, in a way that promotes SCFA production and anti-oxidative and anti-inflammatory actions, thus maintaining a healthy microbiota profile contributing to GI homeostasis. In any case, there is not enough evidence yet to understand the exact type and quantity of individual food components required for effective neuroprotection and which are the exact mechanisms involved. Overall, these data provide a strong rationale for conducting randomized controlled dietary trials in prodromal and manifest PD patients to determine whether a Mediterranean diet can modulate gut microbiota composition and impact disease course [300].

#### 4.5.2. Omega-3 Fatty Acids

In addition to the benefits of SCFAs, several studies have reported that polyunsaturated fatty acids (PUFAs), especially omega (n)-3 (n-3 PUFAs), are essential in the human diet. There are three main types of n-3 PUFAs including eicosapentaenoic acid (EPA), DHA and alpha-linolenic acid (ALA) [301], which are important structural components in cell membranes. N-3 PUFAs are present in high quantities in fish, especially cold-water fatty fish, such as salmon, mackerel, tuna, herring, and sardines; moreover, they can be found as purified supplements [302].

There are many mechanisms by which n-3 PUFAs fatty acids may impact the brain and be beneficial in the prevention and/or treatment of PD. In vivo studies in animal models of PD suggest that n-3 PUFAs can ameliorate motor and cognitive function by partially restoring dopaminergic neurotransmission after 6-OHDA-induced toxicity [303,304,305]. Another study also showed that rotenone-induced motor and non-motor problems were partially alleviated by a therapeutic dietary intervention providing DHA and other nutrients that increased phospholipid synthesis as well as prebiotic fibers [288]. Other studies reported the potential role of n-3 PUFAs as anti-neuroinflammatory agents for the prevention and treatment of PD [306]. Interestingly, n-3 PUFAs treatment could inhibit the damage induced by LPS in rat dopaminergic neurons through the inhibition of NF-κB activation, which is an important factor for microglial activation [307]. Moreover, in a partial lesion model of PD using 6-OHDA, treatment with DHA decreased the astrogliosis and microgliosis both in the striatum and SNpc [308]. Neuroprotective effects on dopaminergic neurons have also been associated with the antioxidant and anti-inflammatory properties of n-3-PUFAs. In this study, n-3-PUFAs supplementation mitigated the loss of dopaminergic neurons in the SNpc and the nerve terminals in the striatum caused by 6-OHDA-induced toxicity in rats [303].

Different clinical studies have also showed the existent relationship between n-3 PUFAs and PD risk. One study found that PD patients had significantly lower n-3 PUFAs intake [309], in agreement with this study, another one found that high n-3 PUFA intake was related to a lower risk of PD [310]. A different double-blind, placebo-controlled pilot study showed that PD patients taking n-3 PUFAs presented improvement in depressive symptoms [311]. Moreover, another study demonstrated that n-3 PUFAs and vitamin E co-supplementation in people with PD had favorable effects on UPDRS and markers of insulin metabolism [312]. In addition, a different randomized clinical trial investigated the effect of n-3 PUFAs supplements on the human gut microbiota and observed that supplementation induced a reversible increase in several SCFA-producing bacteria, including *Bifidobacterium, Roseburia*, *and Lactobacillus* [313]. These observations suggest that n-3-PUFA supplementation may represent an effective way to modify the production of SCFAs and in turn improve GI homeostasis.

Despite the promising data, the development of n-3 PUFA as drugs is restricted by the fact that they are non-patentable compounds, at least in their natural form [314]. In addition, more clinical trials should be carried out by supplementing PD patients with n-3 PUFAs and controlling for all possible co-variates in order to extract definitive conclusions. Nevertheless, their therapeutic potential, favorable safety profile, ease of administration, and low treatment costs are promising [315].

#### 4.5.3. Vitamins

Food rich in vitamins has also gained attention in the treatments and prevention strategies for PD. Since oxidative stress plays an important role in neurodegeneration and PD, vitamins such as vitamin B, C, and E may prevent, delay, or alleviate the clinical symptoms of PD related with oxidative stress, free radical formation, and neuroinflammation [316,317].

Vitamin B3 (i.e., niacin, nicotinamide or nicotinic acid) compounds derive from different food sources, such as beans, meat, fish, milk, mushrooms, and enriched flour [318]. Oral administration of nicotinamide in a MPTP-induced PD mouse model resulted in a dose-dependent sparing of striatal dopamine levels and SN neurons [319]. Another study showed that a nicotinamide supplemented diet rescued mitochondrial impairment and neuroprotected dopaminergic neurons in Drosophila *pink1* mutants, via enhancing the availability of the co-enzyme nicotinamide adenine dinucleotide (NAD^+^) [320]. Interestingly, a recent study showed reduced fecal content of nicotinic acid in PD patients compared with HC [119].

Vitamin C is abundant in vegetables and fresh fruits and vitamin E is abundant in vegetable oils and whole-grain cereals [321]. Despite their antioxidant function, epidemiologic studies investigating associations between these proteins and PD risk, have produced inconsistent and controversial results [322,323,324,325,326]. Thus, additional clinical trials are still needed to confirm the role of vitamins in slowing the progressive deterioration of function in PD and further neurobiological studies to understand how vitamins interact or modulate the gut microbiota. In conclusion, we know that an altered gut microbiota may contribute to the onset or progression of PD; thus, we must acknowledge that some dietary factors may change the gut microbiota in a way that negatively or positively impacts gut homeostasis and directly or indirectly affects PD pathogenesis. Therefore, research to understand the mechanisms by which diet components modulate gut microbiota should be promoted in order to use this knowledge to promote healthy and avoid disease states.

### 4.6. Fecal Microbiota Transplant (FMT)

FMT is the process of delivering fecal material from healthy donors to recipient patients with a disease related to an unhealthy gut microbiome in order to re-establish a stable gut microbiota [327]. FMT has shown a high amount of success in the short-term treatment of *Clostridium difficile* infections, together with low-risk and short-term adverse effects, most commonly bloating, abdominal pain, diarrhea and/or constipation [328,329]. During the last 10 years, there is an increasing interest on the benefits of FMT in GI diseases (i.e., inflammatory bowel syndrome [330]) but also in other diseases where the GI tract is thought to play a role. In the case of neurological disorders, treatments with FMT are limited although there are case reports that it is effective in the treatment of autism [53], multiple sclerosis [331], chronic fatigue syndrome [332], anxiety [333], depression [334], among others.

As mentioned before, pathophysiology in patients with PD and GI non-motor symptoms have been directly linked to gut dysbiosis, increased intestinal permeability and gut inflammation. There is in vivo evidence of FMT studies in mice where an FMT from MPTP-intoxicated mice to non-intoxicated mice caused motor impairments and decreased dopamine levels in the striatum. On the contrary, intoxicated mice receiving FMT from non-intoxicated mice showed an increase of dopamine and 5-HT levels in the striatum along with recovery of motor function [92]. Moreover, the authors showed that FMT reduced the activation of microglia and astrocytes in the SN, as well as it reduced the expression of TLR4/TNF-α signaling pathway components in the colon and the striatum [92]. Another study also carried out FMT from six different PD cases into a mouse model overexpressing α-syn, and they showed that the motor symptoms of the mice were worsened after FMT, suggesting that the altered gut microbiota from PD patients contributes to the development of PD motor symptoms in mice [91]. Based on these reports in experimental mouse models, it seems rational to propose to modify the gut microbiota through long-term FMT administration providing a novel potential therapeutic option for at least some PD patients [23,335]. FMT is an interesting technique as it is relatively simple to apply, and it generally has mild adverse symptoms. However, it will be important to know the microbiota composition of healthy FMT donor samples, as it could be valuable to identify the microbes that may confer beneficial physical and mental effects, as this is still not fully understood [336]. Once we would have this knowledge, we could use these microbes and develop probiotics, thus merging the two techniques to have more effective therapeutic options.

Despite the fact that FMT seems to be a powerful tool to modulate gut microbiota, there is almost no clinical data available for PD. A recent study case on one PD patient who suffered from severe constipation in which improvement of defecation time and reduction of legs tremors was reported one week after FMT treatment [337]. There is another recent preliminary study done with 15 PD patients, where 10 of them received FMT from healthy donors via colonoscopy and five received FMT via nasal-jejunal tube. Results showed that colonic FMT was more effective than nasointestinal FMT. Moreover, patients showed improved motor symptoms evaluated using the UPDRS-III; they improved the quality of their sleep, and anxiety and depression were also partially relieved after three months. However, five cases presented adverse effects including diarrhea, abdominal pain, and flatulence [338]. However, clinical data from only two studies are obviously not sufficient to support the therapeutic value of FMT for PD. There is an on-going double-blind, placebo-controlled randomized clinical trial in which they are studying the effects of FMT administration from healthy donors to PD patients in order to assess the development of the symptoms [339]. We will wait for the results of this clinical trial with high interest. Because PD is a multifactorial disease in which it is still not clear how exactly gut microbiota affects the onset or the progression of the disease, even though FMT is an attractive way to modulate gut microbiota for PD patients, we need time to understand the biological basis of gut microbiota involvement in PD pathophysiology. In addition, it is necessary to have more clinical trials in order to have significant results and to be able to design therapeutic interventions in a standardized way [336,340]. Since current pharmacotherapeutic options are limited for PD patients, in the near future, long-term administration of probiotics and FMT may become a novel non-invasive therapeutic approach to benefit PD patients.

### 4.7. Live Biotherapeutic Products (LBPs)

Apart from the already mentioned classical approaches for modulating gut microbiota (i.e., antibiotics, probiotics, prebiotics, dietary compounds, and FMT), there are new challenges and opportunities in order to modulate the structure and function of gut microbiota from a therapeutic point of view. Microbes can be engineered to act like living therapeutic factories designed and developed to perform specific actions in the human body in order to treat, cure or prevent a disease, infections or disorders [341]. The Food and Drug Administration (FDA) defined LBPs as living organisms, which does not include vaccines, viruses, or oncolytic bacteria, which are applicable to the prevention, treatment, or cure of a disease or condition. LBPs are distinguished from probiotic supplements as most probiotics are regulated as dietary supplements and cannot make claims to treat or prevent disease. However, some probiotics can also fit in the LBP definition. Other LBPs can include recombinant LBPs that are genetically modified organisms that have been engineered by adding, deleting, or altering genetic material within the organism [342].

Recently, an in vitro study provided evidence of two gut bacterial strains, *Parabacteroides distasonis* MR×0005 and *Megasphaera massiliensis* MR×0029, that produced SCFAs and displayed intrinsic antioxidant capacity suggesting they could modulate relevant cell types targeted by neuroinflammation and oxidative stress in neurodegenerative diseases such as PD [343]. These results suggest that LBPs, as opposed to isolated metabolites, have the ability of producing more than one effect, being a novel drug class to consider in drug discovery for PD.

Engineered LBPs have the advantage of being designed in order to perform targeted therapeutic delivery with much greater control of location and timing [344]. Otherwise, those drugs would be rapidly degraded in the bloodstream or during transit of the upper GI tract. They can be equipped with devices for sensing inputs, building memory, and controlling gene expression, such that they can produce and deliver the active compound of interest at a specific time and in a specific location [345]. Recently, a study engineered a strain of *Lactococcus lactis* to evaluate its neuroprotective effect against MPTP toxicity in mice [346]. Their results indicated that oral administration of the engineered LBPs significantly reduced MPTP-induced motor impairment and neuroinflammation [346].

More sophisticated approaches can be created to sense and respond to features of the gut environment; devices can be set up in a way that they can respond to stress, temperature, quorum-sensing signals, and other small molecules [347]. The most recent studies focusing on the gut microbiome for engineering living therapeutics have agreed on a preferred chassis for living factories: *Escherichia coli*, *Bacteroides*, and *lactic acid bacteria* [347,348,349]. One study created an engineered LBP with thiosulfate and tetrathionate sensors that was able to detect gut inflammation and emitted a fluorescent protein signal that could easily be quantified [350]. Another study also engineered LBP to detect tetrathionate, but, in this case, the strain used retained memory of exposure in the gut for analysis by fecal testing over a six-month period [351]. Both studies confirmed the potential of engineered LBPs as living diagnostic tools to detect gut inflammation. Thus, this approach could be used in new studies and replicate it for a wide range of bacterial sensors to enable the creation of a new class of minimally invasive techniques.

The potential for synthetic biology to contribute to PD clinical diagnostic and therapeutic development is really exciting. The ability to generate bacterial strains with unique and increasingly complex functions has rapidly expanded in recent years, although we still need much more fundamental work before we can think of the real impact in preclinical research [352].

## 5. Conclusions and Future Perspectives

**Figure 1:**
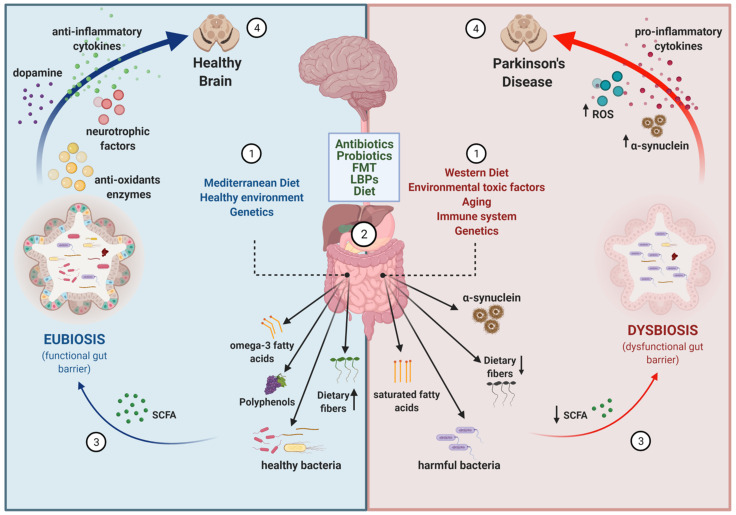
Disease-modifying strategies based on the gut microbiota. (1) The gut microbiota composition is influenced by several intrinsic (i.e., genetics, aging and immune system function) and extrinsic factors, including dietary habits (e.g., Mediterranean diet vs. Western diet) and environmental conditions (e.g., healthy environment vs pesticides/polluted environment). (2) Several strategies can be used to modify the composition of the gut microbiota to revert a dysbiotic condition: antibiotics, probiotics, fecal microbiota transplants (FMT), live biotherapeutic products (LBP), and dietary factors. (3) As a result, the balance between healthy/harmful bacteria, the presence of dietary fibers, the levels of metabolites with anti/pro-oxidant and anti/pro-inflammatory properties (e.g., omega-3 fatty acids, polyphenols, short chain fatty acids (SCFA), saturated fatty acids), and the aggregation of proteins like α-synuclein in the gut will be modified. (4) Because of the existing communication between the gut and the brain, these changes in the gut may have a direct impact on brain function through different mechanisms: levels of neurotransmitters, cytokines, reactive oxygen species (ROS), neurotrophic factors, and aggregation of α-synuclein. Overall, changes of the gut microbiota composition can have a beneficial or detrimental impact on the neurodegenerative process occurring in Parkinson’s disease.

Unprecedented breakthroughs in microbiome research have been achieved in the past 15 years. In line with this exciting new field in biomedical research, the gut microbiota has become an increasingly attractive research area in the quest to better understand the pathogenesis of PD and several studies have shown that PD patients have abnormal gut microbiota. Current studies should aim at clarifying the role of gut dysbiosis in PD development and progression. The future looks promising as we begin to recognize the specific strains and mechanisms underlying the effects of gut dysbiosis, and, as we see, the first attempts of clinical application in PD (Figure 1). However, there are still major limitations and challenges that the research community needs to address, especially regarding the definition of those specific strains that are directly related to PD pathogenesis independently of other co-variables (i.e., age, sex, ethnicity, geography, diet, lifestyle, medication), the mechanistic understanding of host–microbiome interactions and the integration of these insights into routine clinical practice. Then, mechanistically focused microbiome research aimed at demonstrating causality rather than association or correlation is a must before microbiome-derived data can really be incorporated into precision medicine.

With this in mind, the opportunities microbiome research is offering the field are extraordinary. First, gut microbiota might represent a unique source for the development of pathophysiology-based therapies. If gut dysbiosis contributes to neurodegeneration and the manifestation of PD-symptoms, a therapeutic approach aiming to reestablish a healthy microbiota seems appropriate. Different strategies can be envisioned: lowering the abundance of certain pathogenic or detrimental strains with selective antimicrobials, repopulating the gut microbiome with alternate bacterial strains (such as with probiotics), or exploring the potential roles of selective FMT. Although preclinical and clinical evidence on these beneficial effects in PD is still very limited, examples from other neurological diseases are encouraging [353,354,355,356]. In addition, the interest in FMT is exemplified by more than 350 completed or planned clinical trials (NIH, December 2020). However, yet again, we need to identify the mechanisms underlying the reported effects of FMT and probiotics in order to move towards more controllable and potent precision interventions. In addition, defining the right time for intervention and the appropriate regime (continuous use vs long-term effects) will also be major challenges in the next few years.

Second, gut microbiota might represent a unique source for biomarkers relevant to early/prodromal disease phases, to predict the in vivo L-Dopa bioavailability and efficacy, and to assess the impact of therapeutic approaches in PD patients. Overall, biomarkers that ultimately will improve the management of the disease. However, for metagenomic studies to be meaningful, the research community should standardize study designs in order to minimize variations due to different sources of technical and biological variability. Moreover, sequencing platforms are currently not available in all medical centers and their implementation into clinical care will require some efforts in sample processing and data analysis to render them clinically interpretable and affordable. Research on additional biomarkers related to gut microbiota dysbiosis not relying on abundance of phyla/families/genera/species of gut bacteria, hold great promise. In this sense, integrated multiomic analyses for functional interrogation of microbiome–host interactions will be very revealing. To have objective measures, a part from clinical scales and questionnaires, to assess the safety and efficacy of different therapeutic interventions in clinical trials, is fundamental.

Finally, gut microbiota might represent a new approach for personalized medicine. Recent studies identifying the bacteria that metabolize L-Dopa to dopamine (*Enterococcus faecalis*) and m-tyramine (*Eggerthella lenta*) have highlighted the benefits of screening patients in order to anticipate those with a deleterious metabolism of L-Dopa in the gut and treat them with small molecule inhibitors to prevent TDC-dependent L-Dopa decarboxylation in the gut. Such discoveries offer a unique potential to personalize L-Dopa treatment in the hope of ameliorating the variability of L-Dopa bioavailability and motor fluctuations. In addition, the field of machine learning (ML) offers unprecedented opportunities for the field of PD. ML includes the development and application of computer algorithms that improve with experience, thus representing appropriate tools to build predictive models for the classification of biological data and identify biomarkers through a training procedure [357]. A recent study processed 846 16S rRNA microbiota published datasets coming from six different studies and applied an ML approach to define a classifier that could predict the pathological status of PD patients against HC. Moreover, they identified a subset of 22 bacterial families that were discriminative for the prediction, and, interestingly, not all families identified by the algorithm were reported in the literature. The identification of new bacterial families that may play an important role in predicting PD status highlights the power of a prediction analysis based on ML algorithms [358]. Importantly, the success of this type of approach is dependent on the willingness of the research community to apply honest data sharing policies.

In conclusion, albeit further studies are needed to clarify the mechanisms of gut dysbiosis in PD pathogenesis and to better define the clinical outcomes and safety issues, the potential usage of microbiota-based therapeutic strategies in treating GI alterations, and possibly also motor symptoms, is definitely in the spotlight, and we will be expectant to the new data coming from microbiome studies and clinical trials.

## Figures and Tables

**Table 1:** Different abundant taxa between Parkinson’s disease (PD) patients and healthy controls (HC).

Phylum	Family	Genus	Increased Abundance	Decreased Abundance	References
*Actinobacteria*			5	0	[99,103,115,117,119]
*Actinobacteria*	*Bifidobacteriaceae*		5	0	[98,103,104,107,119]
*Actinobacteria*	*Bifidobacteriaceae*	*Bifidobacterium*	6	2	[95,97,98,100,102,107,111,119]
*Bacteroidetes*			2	5	[95,97,99,101,104,109,119]
*Bacteroidetes*	*Prevotellaceae*		0	5	[24,60,97,105,109]
*Bacteroidetes*	*Prevotellaceae*	*Prevotella*	3	5	[24,60,95,98,100,107,110,111]
*Firmicutes*			3	4	[60,101,103,104,113,115,117]
*Firmicutes*	*Enterococcaceae*		3	1	[96,97,99,106]
*Firmicutes*	*Lachnospiraceae*		0	9	[98,101,103,104,106,107,117,118,119]
*Firmicutes*	*Lachnospiraceae*	*Roseburia*	0	10	[98,101,103,106,107,111,114,117,118,119]
*Firmicutes*	*Lachnospiraceae*	*Blautia*	0	6	[95,98,99,101,111,119]
*Firmicutes*	*Lactobacillaceae*		5	1	[96,97,98,103,106,117]
*Firmicutes*	*Lactobacillaceae*	*Lactobacillus*	5	1	[95,98,100,102,110,111]
*Firmicutes*	*Ruminococcaceae*		3	2	[24,98,99,109,117]
*Firmicutes*	*Ruminococcaceae*	*Faecalibacterium*	0	10	[95,97,98,99,104,111,112,114,117,118]
*Proteobacteria*			4	0	[99,101,103,119]
*Proteobacteria*	*Enterobacteriaceae*		6	0	[24,97,99,103,104,106]
*Verrucomicrobia*			6	0	[101,103,105,113,115,119]
*Verrucomicrobia*	*Verrucomicrobiaceae*		8	0	[60,98,101,103,105,106,109,119]
*Verrucomicrobia*	*Verrucomicrobiaceae*	*Akkermansia*	13	0	[60,97,98,101,103,105,109,110,113,114,115,118,119]

List of the most frequently found, at least in four independent cohorts, reported fecal microbiota alterations between PD patients and HC. Reported taxa are indicated in bold and higher taxonomic levels are provided when applicable. The number of publications reporting an increase or a decrease in the relative abundance in PD patients are indicated. Adapted from Boertien et al. [49].

**Table 2:** Neuroprotective effects of antibiotics in different experimental PD models.

Antibiotic	Neuroprotection	References
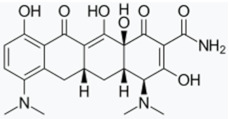 **Minocycline**	• Modulates MPTP/6-OHDA-induced microglia activation: o Inhibits p38 MAPK activation o Decreases the release of cytokines o Decreases iNOs activation, thus reducing the production of NO • Inhibits caspase-1 expression	[210,211,212,213,214]
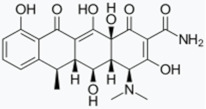 **Doxycycline**	• Modulates MPTP/6-OHDA-induced microglia and astrocyte activation: o Decreases iNOs activation, thus reducing the production of NO o Downregulation of MMP-3 activity • In vitro biochemical assay: reshapes α-syn oligomers towards non-toxic structures	[202,218,219,220,221]
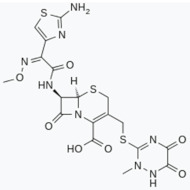 **Ceftriaxone**	• Modulates MPTP/6-OHDA-induced toxicity: oIncreases GLT-1 expression oPrevents MPTP-induced motor impairment • Reduces pro-inflammatory cytokine and factors: TNF-α and IL-β • Restores the levels of endogenous antioxidant enzymes • In vitro biochemical assay: blocks α-syn polymerization	[222,223,225,226,227]
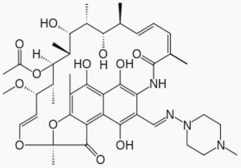 **Rifampicin**	• Modulates rotenone-induced toxicity: o Upregulates GRP78 via the PERK-eIF2α-ATF4 pathway • Modulates rotenone-induced microglial activation: o Downregulates NF-kB and MAPKs pathways o Decreases the release of pro-inflammatory cytokines and factors: TNF-α, IL-β and IL-6 o Decreases iNOs activation, thus reducing the production of NO • Activates the autophagy pathway • Diminishes oxidative stress induced by MPTP toxicity • In vitro biochemical assay: inhibits α-syn fibrillation and aggregation	[228,229,230,231,232,233,234,235]

BDNF: brain-derived neurotrophic factor. GDNF: glial cell-derived neurotrophic factor. GLT: glutamate transporter subtype 1. GRP78: glucose-regulated protein 78. IL: interleukin. iNOs: inducible nitric oxide synthase. MAPK: mitogen-activated protein kinase. MMP: matrix metalloproteinase 3. MPTP: 1-methyl-4-phenyl-1,2,3,6-tetrahydropyridine. NF-kB: nuclear factor-kappa B. NO: nitric oxide. PD: Parkinson’s disease. ROS: reactive oxygen species. TNF- α: tumor necrosis factor alpha. 6-OHDA: 6-hydroxydopamine. α-syn: alpha-synuclein.

**Table 3:** Neuroprotective effects of probiotics on pathological features of PD.

Probiotic Type	Concentrations	Treatment Duration	Tested in	Main Results	References
*Lactobacillus salivarius*, *Lactobacillus plantarum*, *Lactobacillus acidophilus*, *Lactobacillus rhamnosus*, *Bifidobacterium animalis* subsp. *lactis*, *Bifidobacterium breve*	X	Co-culture with probiotic	In vitro. Peripheral blood mononuclear cells from PD patients.	• Decrease of pro-inflammatory cytokines • Increase of anti-inflammatory cytokines • Reduced ROS production	[244]
*Lactobacillus salivarius*, *Lactobacillus plantarum*, *Lactobacillus acidophilus*, *Lactobacillus rhamnosus*, *Bifidobacterium animalis* subsp. *lactis*, *Bifidobacterium breve*	X	1h probiotic exposure or 1 h probiotic + inflammatory stressor	In vitro. Caco-2 cell line	• Restored the integrity of the epithelial damaged cells	[244]
*Lactobacillus salivarius*, *Lactobacillus plantarum*, *Lactobacillus acidophilus*, *Lactobacillus rhamnosus*, *Bifidobacterium animalis* subsp. *lactis*, *Bifidobacterium breve*	X	48 h	In vitro. *Escherichia coli* and *Klebsiella pneumoniae* inoculation	• Inhibited the growth of potential pathogen bacteria	[244]
*Bacillus subtilis*	X	13 days	*In vivo. Caenorhabditis elegans* (C. *elegans*) model of synucleinopathy	• Inhibited α-syn aggregation • Cleared preformed aggregates	[245]
*Lacobacillus rhamnosus* GG, *Bifidobacterium animalis lactis* and *Lactobacillus acidophilus*	2 × 10^6^ CFU	1X a day for 4 weeks	*In vivo. MPTP-induced mouse model and Rotenone-induced mouse model.*	• Neuroprotective effects on dopaminergic cells against MPTP and Rotenone-induced toxicity: o Upregulate expression of BDNF and GDNF o Downregulate expression of MAO-B o Increase levels of butyrate • Prevented behavioral impairment	[246]
*Lactobacillus plantarum PS128*	1 × 10^9^ CFU	1X a day for 28 days	*In vivo. MPTP-induced mouse model*	• Neuroprotective effects on dopaminergic cells against MPTP induced toxicity: o Upregulates expression of BDNF o Decreases the expression of pro-inflammatory cytokines o Reduces glial reactivity • Improved behavioral impairment	[248]
*Clostridium butyricum*	5 × 10^8^ CFU	1X a day for 4 weeks	*In vivo. MPTP-induced mouse model*	• Neuroprotective effects on dopaminergic cells against MPTP induced toxicity: o Improved microglia activation and synaptic dysfunction o Increased levels of colonic GLP-1 and GLP-1 receptor in the brain. • Improved behavioral impairment	[249]
*Bifidobacterium bifidum*, *Bifidobacterium longum*, *Lactobacillus rhamnosis*, *Lactobacillus rhamnosus* GG, *Lactobacillus plantarum* and *Lactococcus lactis*	1 × 10^10^ CFU	1X a day for 16 weeks	*In vivo. MitoPark mouse model*	• Attenuated motor impairment • Reduced dopaminergic cell loss	[252]
*Lactobacillus acidophilus, Bifidobacterium bifigum, Lactobacillus reuteri and Lactobacillus fermentum (capsules)*	8 × 10^9^ CFU	1X a day for 12 weeks	*PD patients*	• Decreased MDS-UPDRS scores	[253]
*Lactobacillus casei Shirota* (fermented milk)	6.5 × 10^9^ CFU	1X a day for 5 weeks	*PD patients*	• Improved stool consistency • Reduced bloating and abdominal pain	[145]
*Streptococcus salivarius,* subsp. *Thermophilus, Enterococcus faecium, Lactobacillus rhamnosus* GG, *Lactobacillus acidophilus, Lactobacillus plantarum, Lactobacillus paracasei, Lactobacillus delbrueckii,* subsp. *Bulgaricus* and *Bifidobacterium* (fermented milk) + Prebiotic fiber	2.5 × 10^11^ CFU	1X a day for 4 weeks	*PD patients*	• Improved constipation symptoms	[250]
*Lactobacillus acidophilus, Lactobacillus reuteri, Lactobacillus gasseri, Lactobacillus rhamnosus, Bifidobacterium bifidum, Bifidobacterium longum, Enterococcus faecalis, Enterococcus faecium* (capsules)	1 × 10^10^ CFU	1X a day for 4 weeks	*PD patients*	• Improved constipation symptoms	[251]
*Lactobacillus acidophilus* and *Bifidobacterium infantis* (tablets)	120 mg/day X	2X a day for 12 weeks	*PD patients*	• Reduced bloating and abdominal pain	[146]

BDNF: brain-derived neurotrophic factor. CFU: colony-forming unit. GDNF: glial cell-derived neurotrophic factor. MAO-B: monoamine Oxidase-B. MDS-UPDRS: The Movement Disorder Society-Unified Parkinson’s Disease Rating Scale. MPTP: 1-methyl-4-phenyl-1,2,3,6-tetrahydropyridine. PD: Parkinson’s disease. ROS: reactive oxygen species. α-syn: alpha-synuclein. X: CFU was unavailable.

## Data Availability

No new data were created or analyzed in this study. Data sharing is not applicable to this article.
